# Gestational Age-Dependent Interplay between Endocannabinoid Receptors and Alcohol in Fetal Cerebral Arteries

**DOI:** 10.4303/jdar/236068

**Published:** 2019

**Authors:** Maria Simakova, Ana Tobiasz, Ryan D Sullivan, Shivantika Bisen, Jose Duncan, J. Pierce Sullivan, Steven Davison, Danielle L Tate, Stacey Barnett, Giancarlo Mari, Alex M Dopico, Anna N Bukiya

**Affiliations:** 1 Department of Pharmacology, University of Tennessee Health Science Center, Memphis, TN, USA; 2 Department of Obstetrics and Gynecology, University of Tennessee Health Science Center, Memphis, TN, USA; 3 Department of Comparative Medicine, University of Tennessee Health Science Center, Memphis, TN, USA

**Keywords:** prenatal alcohol, fetal cerebral artery, maternal alcohol consumption, cannabinoid system, cannabinoid receptor

## Abstract

Alcohol (ethanol) is one of the most widely consumed drugs. Alcohol consumption by pregnant women may result in a range of fetal abnormalities termed fetal alcohol spectrum disorders (FASDs). The cerebrovascular system is emerging as a critical target of alcohol in the developing brain. We recently showed that three episodes of prenatal alcohol exposure resulting in 80 mg/dL alcohol in maternal blood during mid-pregnancy up-regulated anandamide-induced dilation of fetal cerebral arteries. Moreover, ethanol dilated fetal cerebral arteries via cannabinoid (CB) receptors. Whether a critical role of fetal cerebral artery CB system in responses to alcohol was maintained throughout the gestation, remains unknow.

**Main methods.:**

Pregnant baboons (second trimester equivalent) were subjected to three episodes of either alcohol or control drink infusion via gavage. Cerebral arteries from mothers and near-term female fetuses were *in vitro* pressurized for diameter monitoring.

**Key findings.:**

Near-term fetal and maternal arteries exhibited similar ability to develop myogenic tone, to constrict in presence of 60 mM KCl, and to respond to 10 µM anandamide. Fetal and maternal arteries largely failed to dilate in presence of 63 mM ethanol. No differences were detected between arteries from control and alcohol-exposed baboon donors. Therefore, previously observed ethanol-induced dilation of fetal cerebral arteries and up-regulation of CB components in response to fetal alcohol exposure during mid-pregnancy was transient and disappeared by near-term.

## 1. Introduction

Alcohol (ethanol) is one of the most widely consumed drugs worldwide [1]. Prenatal alcohol exposure (PAE) can lead to a range of developmental abnormalities termed Fetal Alcohol Spectrum Disorder (FASDs) [2]. The global prevalence of FASDs among children and youth is estimated to be 7.7 per 1,000 people [3]. In the US, FASDs represents the leading preventable cause of neurodevelopmental disability [4]. Fetal cerebrovascular system is currently emerging as a critical target for alcohol in the developing brain [5,6,7,8,9].

Studies in murine and non-human primate species consistently point at increased fetal cerebral perfusion following maternal alcohol intoxication [5,6,7]. In an ovine model, larger alterations in fetal cerebral blood flow by alcohol were linked to the severity of subsequent neuronal loss [10]. Our recent study showed that alterations in baboon fetal cerebral artery indices in response to maternal alcohol consumption during second trimester equivalent preceded morphometric developmental delays [11]. Thus, the evidence strongly suggests that alcohol-driven modifications of cerebrovascular function play a critical role in the detrimental effect of maternal drinking on fetal development. However, the molecular targets of ethanol within fetal cerebral arteries are largely unknown.

The endocannabinoid (eCB) system includes eCB receptors, their endogenous ligands (such as anandamide), and metabolic enzymes involved in eCB synthesis and degradation [12]. Using a baboon model, we have recently established presence of cannabinoid receptor type 1 (CB1) and 2 (CB2) in fetal cerebral artery lysates during mid-pregnancy [8]. Moreover, we detected an increase in cannabinoid receptor type 2 (CB2)-mediated dilation of fetal cerebral arteries by anandamide in fetuses that were subjected to repeated alcohol exposure during the second trimester equivalent [8]. Whether up-regulation of CB2 receptor function during mid-pregnancy constitutes a transient response or rather, results in a long-term adaptation of the eCB system to alcohol exposure remains unknown.

In the present work, we utilized a baboon model of maternal alcohol consumption and selective pharmacology on *in vitro* pressurized fetal and maternal cerebral arteries to determine whether PAE during the second trimester equivalent has long-lasting effects on fetal cerebral artery eCB system. During PAE episodes, maternal blood alcohol level (BAL) reached 80 mg/dL (≈17 mM). This concentration represents the legal limit of alcohol intoxication for driving a motor vehicle in most of the United States. While lethal BALs in human average 355 mg/dL [13], BAL in our study constitutes a rather modest and clinically relevant amount. Considering the sex differences in CB pharmacology [14,15], we focused on cerebral arteries of female fetuses and their maternal counterparts.

We determined that eCB system contribution into ethanol targeting of fetal cerebral artery diameter was limited to mid-pregnancy and disappeared by near-term. Our findings point at a gestational stage-dependent effect of ethanol on fetal cerebral artery diameter.

## 2. Material and Methods

### 2.1. Ethics and study approval

The care of animals and experimental protocols were reviewed and approved by the Animal Care and Use Committee of the University of Tennessee Health Science Center, which is an Association for Assessment and Accreditation of Laboratory Animal Care International (AAALACi)-accredited institution.

### 2.2. Animal subjects

Baboons were received from the University of Oklahoma Primate Research Center. Gestational age of candidate baboons for the study was determined by this Center based on female baboon cycles and confirmed with ultrasound examination. Before reaching 80 days of gestation, baboon dams were transported to the University of Tennessee Health Science Center (UTHSC) and given ten days to acclimate to the new environment. Dams were alcohol-naïve but had been used in other research studies. Baboons were housed at UTHSC as detailed in our recent work [8]. Briefly, dams were singly housed in standard baboon cages, with visual and audio access to each other. A maximum of four baboons (cages) were housed per room, on a 12 h light/dark cycle (lights on at 6:00 am). Baboons were fed twice a day, each feeding consisting of the High Protein Monkey Diet (~15 biscuits per meal, 21 kcal/biscuit) to sustain a baboon’s weight gain as expected throughout the pregnancy. Each feeding was also supplemented by two pieces of fresh fruits or vegetables and two tablespoons of peanut butter. Drinking water was available *ad libitum*. The facilities were maintained in accordance with the USDA and AAALACi standards. Species-appropriate toys, video presentations, and human interactions were provided to dams on a daily basis as means of environmental and social enrichment. Housing conditions were consistent throughout the study.

### 2.3. Confirmation of gestational age and alcohol infusion procedure

Dams were subjected to either alcohol or control infusion procedures at 90, 100 and 110 days of gestation. On the day of the first procedure (90 ± 5 days of gestation), animals were anesthetized (see below) and gestational age was once again confirmed by sonography using SonoAce R3 by Samsung (Seoul, South Korea). Fetal abdominal circumference, head circumference and femur length were measured in millimeters and compared to commonly accepted fetal baboon growth curve parameters to verify the gestational age. The study involved a total of seven *Papio hamadryas anubis* dams (ages 7–15 yrs; 90 ± 5 days of gestation) carrying single female fetuses. Dams were randomly assigned to the experimental groups: three dams were receiving control infusions, while four dams were receiving alcohol-containing ones. For the experimental group, the infusion contained 1.8 g/kg ethanol (ultrapure, 200 proof; American Bioanalytical, Natick, MA) diluted in reverse osmosis purified drinking water. This alcohol dose rendered on average 80 mg/ dL alcohol in maternal blood and 63 mg/dL alcohol in amniotic fluid [8]. The control group of animals received an iso-caloric solution containing orange-flavored Tang® powder (Kraft Foods; Northfield, IL). In both cases, the total volume of the drink was equal to 200 mL. Prior to each infusion procedure, the animals were fasted for 12 h. On the day of the infusion, the animal sedation was induced by a single injection of ketamine hydrochloride (Ketaset, 10 mg/kg IM). Throughout the infusion, anesthesia was maintained with isoflurane (1.5–2.0%) in oxygen. Animal monitoring of vital signs and depth of anesthesia consisted of electrocardiography, pulse oximetry, capnography, non-invasive blood pressure and temperature measurements. A rectal suppository of indomethacin (25 mg) was used as preterm labor prophylaxis during the procedure. An orogastric tube was introduced into the stomach and the infusion was administered over 10 minutes. Both groups of animals received a single injection of carprofen (Rimadyl, 4.4 mg/kg IM) aimed to alleviate symptoms of hangover following alcohol drinking. Cesarean deliveries were performed at 165 ± 5 days of gestation. This gestational age represents near-term, as term pregnancy in baboons is described as 175–185 days of gestation During c-section, fetuses were euthanized by exsanguination while still under maternal anesthesia and subsequently administered Euthasol (0.5 mL per fetus IV). While still anesthetized, mothers were euthanized by infusion of Euthathal (1 ml/10 lbs IV). Fetal and maternal brain tissue was immediately harvested for cerebral artery dissection and pressurization.

### 2.4. Pressurized fetal cerebral artery diameter monitoring

Artery diameter monitoring was performed following a general methodology described in our previous work [8]. An arterial segment from the middle cerebral artery (MCA) territory was dissected out of the brain and cannulated at each end in a temperature-controlled, custom-made perfusion chamber using a Dynamax RP-1 peristaltic pump (Rainin Instruments, Inc., Oakland, CA). The chamber was continuously perfused at a rate of 3.75 ml/min with physiologic saline solution (PSS) (mM): 119 NaCl, 4.7 KCl, 1.2 KH_2_PO_4_, 1.6 CaCl_2_, 1.2 MgSO_4_, 0.023 EDTA, 11 glucose, and 24 NaHCO_3_. The PSS was equilibrated at pH 7.4 with a 21/5/74% mix of O_2_/CO_2_/N_2_ and maintained at 35–37°C. Arteries were observed with a CCD camera (Sanyo VCB-3512T; Sanyo Electric Co., Moriguchi, Japan) attached to an inverted microscope (Nikon Eclipse TS100; Nikon). The artery external wall diameter was measured using the automatic edgedetection function of IonWizard software (IonOptics, Milton, MA) and digitized at 1 Hz.

Steady-state changes in intravascular pressure were achieved by elevating an attached reservoir filled with PSS and were monitored using a pressure transducer (Living Systems Instruments, Burlington, VT). Arteries were first incubated at an intravascular pressure of 10 mm Hg for 10 min. Then, arteries were pressurized at 30 mmHg as this pressure has been used by our group in studies on fetal baboon cerebral arteries [8]. Intravascular pressure was held at 30 mmHg throughout the experiment to develop and maintain the arterial myogenic tone. Tone was determined as a percent difference between the maximal diameter reached during application of 30 mmHg intraluminal pressure and steady-state diameter 30 min after pressure application. At the beginning of the experiment, arteries were briefly probed by 60 mM KCl solution to evoke vasoconstriction and verify viability of the artery segments. Drugs were dissolved to make stock solutions, diluted in PSS to their final concentration, and applied to the artery *via* chamber perfusion. The effect of a drug application was evaluated at the time it reached a maximal, steady level.

### 2.5. Quantitative RT-PCR

Total RNA was isolated from frozen middle cerebral artery and their branches of baboon mothers and near-term fetuses by using QIAcube (Qiagen, Hilden, Germany) at UTHSC Molecular Resource Center on a fee-for-service basis. Complementary DNA was synthesized from isolated RNA by The High capacity cDNA Reverse Transcription kit (Applied Biosystems, Foster City, CA) according to manufacturer’s instructions. The cDNA was pre-amplified using TaqMan PreAmp master mix kit (Applied Biosystems, Foster City, CA) by using same amount of total cDNA in each sample. Kappa Probe Fast qPCR master mix(2x) was used to run PCR and expression of gene of interest was measured by TaqMan quantitative PCR using LC480 cycler (Roche Life Science, Penzberg, Germany) at UTHSC MRC facility on a fee-for-service basis. TaqMan probes and primers for genes encoding CB1, CB2, and actin are specified in our recent publication [8].

### 2.6. Chemicals

Alcohol (ethyl alcohol, 100% purity, 200 proof) was purchased from American Bioanalytical (Natick, MA). Anandamide (AEA), Tocrisolve, AM251, and AM630 were purchased from Tocris Bioscience (Bristol, United Kingdom). All other chemicals were purchased from Sigma-Aldrich (St. Louis, MO).

### 2.7. Data analysis

Final plotting, fitting, and statistical analysis of data were conducted using Origin 8.5.1 (OriginLab; Northampton, MA) and InStat 3.0 (GraphPad; La Jolla, CA). Statistical analysis was conducted using two-way analysis of variance (ANOVA) to compare datasets across age (fetus versus adult) and treatment group (control versus alcohol-exposed). We recognize that the use of two-way ANOVA on datasets with small number of observations increases the chances of a Type1 statistical error. However, our data did not yield any statistically significant differences between the groups (see Results and Discussion). Remaining datasets were analyzed using either non-paired Student’s t test (when Gaussian distribution of data was confirmed by InStat software) or by Mann-Whitney nonparametric test when number of observations was low to ensure that the dataset followed Gaussian distribution. For comparison of anandamide-induced dilation of maternal arteries, as well as ethanol-induced dilation of fetal or maternal arteries, results were evaluated by both Mann-Whitney and unpaired t-test, as latter was reported to be suitable for comparison of groups with extremely low sample size [17]. We refrained from using paired comparisons, because one fetus did not have matching maternal data. P<0.05 was considered statistically significant. Data are expressed as the mean ± S.E.M., and n=the number of artery segments. When reported “n” exceeds number of animal donors, no more than two artery segments were collected from each donor. On several occasions, artery segments lost their tone and responsiveness to pharmacological stimuli during the experiment. These artery segments were excluded from the analysis, leaving “n” lower than number of animal donors. For exact number of observations in each case, please refer to the “n” in the corresponding section of the manuscript.

## 3. Results

### 3.1. Fetal and maternal arteries exhibit similar functional characteristics

**Figure 1:**
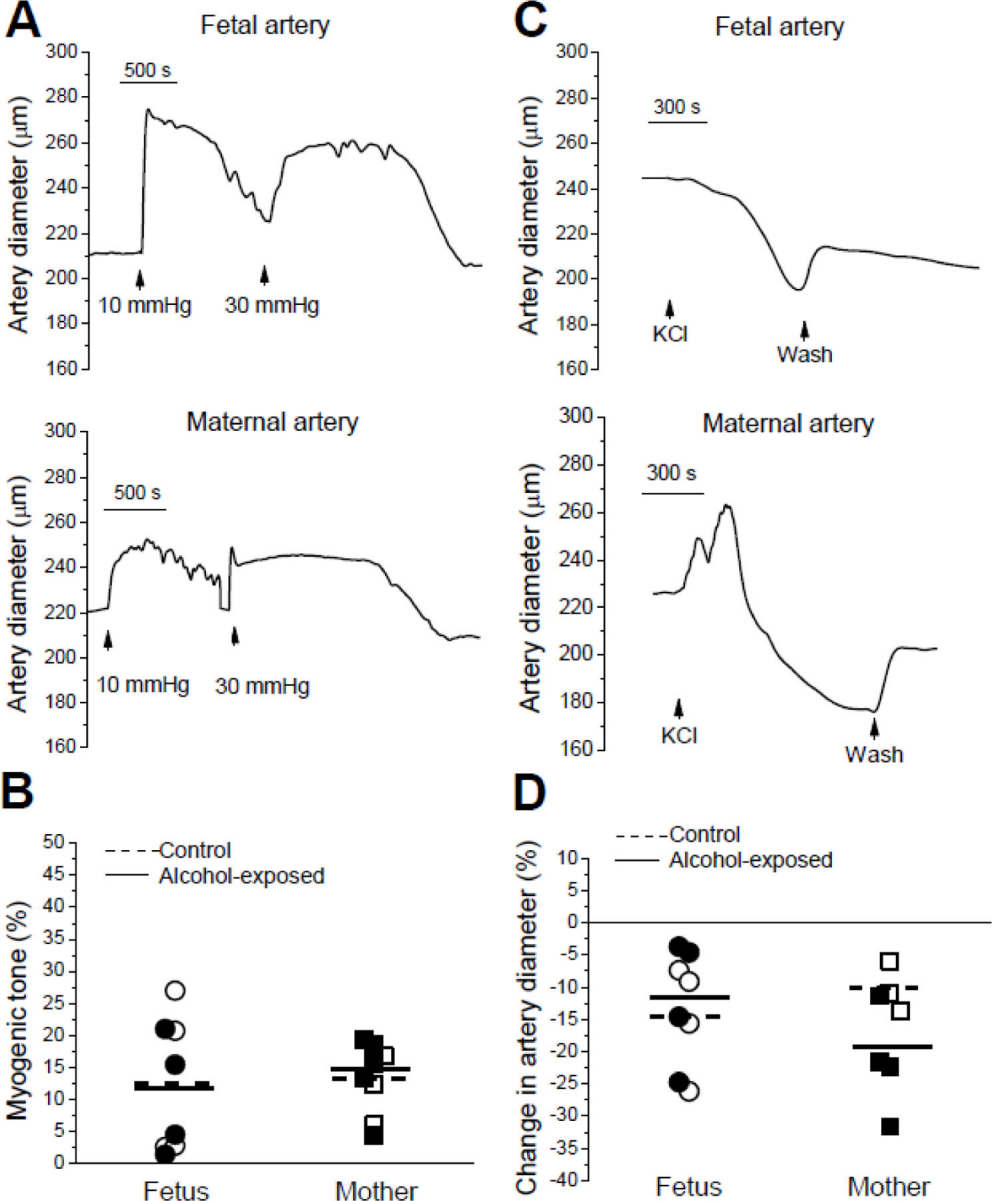
Myogenic tone and responses to high KCl in fetal and maternal arteries. A. Original traces showing development of myogenic tone in fetal (top) and maternal (bottom) arteries following application of 10 and 30 mmHg intraluminal pressure. B. Scattered data of myogenic tone as percentage of maximal artery diameter immediately following application of 30 mmHg intraluminal pressure in fetal and maternal arteries. In all figures, dotted horizontal line depicts averaged of control groups (fetal or maternal), while solid line corresponds to averaged value in alcohol-exposed groups (fetal or maternal); hollow symbols refer to datapoints from control donors, black symbols represent data from alcohol-exposed donors. Statistical analysis using two-way ANOVA failed to detect significant differences or interactions between the groups at the 0.05 level (P values≥0.58). C. Original traces showing constriction of fetal (top) and maternal (bottom) arteries following application of 60 mM KCl. D. Scattered data of KCl-induced constriction as percent change in artery diameter show lack of statistically significant differences between the groups at the 0.05 level (two-way ANOVA, P≥0.13).

First, we compared myogenic tone in fetal arteries from a control group to cerebral arteries from fetuses that were exposed to maternal alcohol during the period equivalent to the second trimester of human pregnancy to determine whether fetal alcohol exposure had long-lasting effects on autoregulation of cerebral arteries. Averaged myogenic tone in the control group (n=5) reached 12.04 ± 4.99% and did not differ significantly from tone in arteries from alcohol-exposed fetuses (n=4), which reached on average 10.52 ± 4.61% (P=0.90 by Mann-Whitney test; Figure 1A–B). In maternal arteries, averaged myogenic tone in the control group (n=4) was 12.66 ± 2.42% and did not differ significantly from tone in maternal arteries from alcohol-exposed donors (n=5), which reached on average 14.35 ± 2.71% (P=0.56 by Mann-Whitney test; Figure 1A–B). A two-way ANOVA failed to detect any interaction between age or alcohol exposure variances (P=0.69). Following myogenic tone development, fetal and maternal arteries were probed with 60 mM KCl to determine the contractile capability of the artery segments (Figure 1C). In most cases, artery diameter remained lower after KCl washout when compared to the pre-KCl level (Figure 1C). Thus, we averaged the pre-and post-KCl diameters and used this value as a baseline to quantify percentage change in an artery diameter by KCl. Averaged constriction by KCl in control fetal arteries (n=4) reached 14.58 ± 4.25% and did not differ significantly from the response to KCl in arteries from alcohol-exposed fetuses (n=4), which reached on average 11.93 ± 4.93% (P=0.49 by Mann-Whitney test; Figure 1C–D). The KCl-induced diameter response in maternal arteries was often biphasic: we observed initial dilation followed immediately by constriction (Figure 1C, bottom trace). This biphasic effect has been often observed in adult cerebral arteries from other species [18–19] (see Discussion). In maternal arteries, the average constriction by KCl in the control group (n=3) was 10.22 ± 2.27% while KCl-induced constriction in maternal arteries from the alcohol-exposed donors (n=4) reached on average 21.75±4.14% (Figure 1C–D). The apparent difference did not reach statistical significance between these groups with the available number of artery segments (P=0.11 by Mann-Whitney test). A two-way ANOVA failed to detect any interaction between age or alcohol exposure variances (P=0.13).

Thus, alcohol exposure did not affect two basic arterial properties: ability to develop myogenic tone and to constrict to a depolarizing agent (KCl). Moreover, there was no difference in myogenic tone and KCl responses between fetal and maternal cerebral arteries.

### 3.2. Pharmacological probing of CB receptor function reveals both commonalities and differences between fetal and maternal cerebral arteries

**Figure 2:**
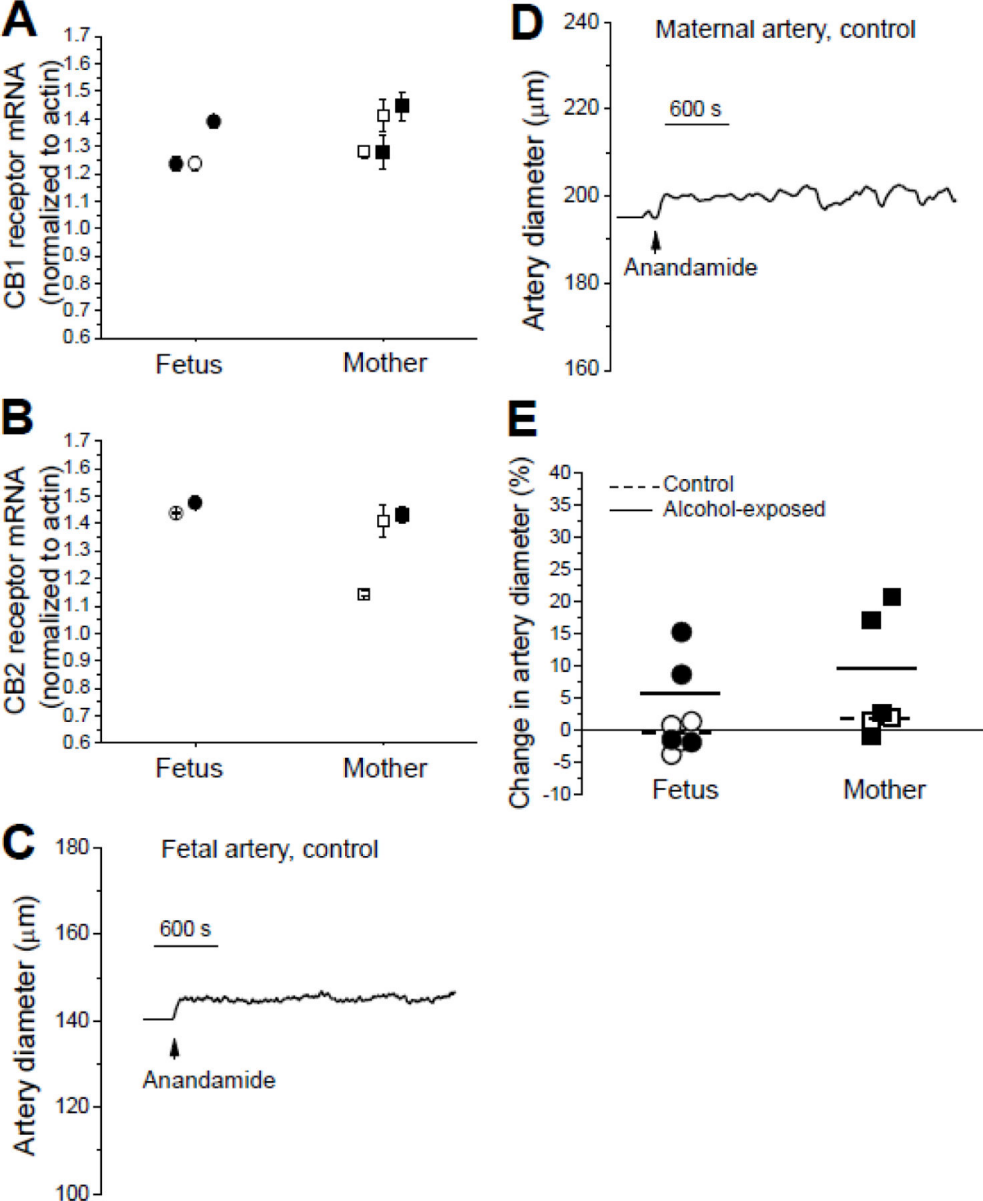
Fetal and maternal cerebral artery responses to anandamide. A. CB1 receptor mRNA level normalized to actin in maternal and near-term fetal cerebral artery lysates. Each datapoint represents an averaged from 4 technical replicates from the same animal donor. Here and in B, error bars represent standard deviations of those 4 technical replicates for each animal donor. B. CB2 receptor mRNA level normalized to actin in maternal and near-term fetal cerebral artery lysates. C. Original record of diameter showing anandamide-induced dilation of fetal cerebral artery. D. Original record of diameter showing anandamide-induced dilation of maternal cerebral artery. E. Scattered data showing changes in artery diameter by 10 µM anandamide in fetal and maternal arteries. Statistical analysis using two-way ANOVA failed to detect significant differences or interactions between the groups at the 0.05 level (P values≥0.12).

In our recent study we demonstrated that fetal alcohol exposure during the second trimester equivalent resulted in up-regulated responses to anandamide when cerebral arteries were harvested from fetuses at the end of this period. In the current work, baboons were subjected to the same alcohol exposure paradigm, yet fetal cerebral arteries were harvested at near-term. First, we established presence of CB1 and CB2 receptor mRNA in maternal and near-term fetal cerebral artery lysates. Similar to findings in fetal arteries during mid-pregnancy, fetal arteries near-term and their maternal counterparts contained detectable level of CB1 and CB2 receptor gene transcript (Figure 2A–B). For CB1 receptor, transcript was detected in 7 out of 7 animal donors, while CB2 receptor gene transcript was detected in 5 out of 7 animal donors. Thus, we next proceeded to cerebral artery *in vitro* pressurization (see above) and probing with CB receptor agonist 10 µM anandamide. At this concentration, fetal cerebral artery dilation at the end of mid-pregnancy reached near-maximum level [8]. First, the artery diameter was equilibrated for 15 min in Tocrisolve™- containing solution, this agent serving as a vehicle for anandamide. Then, responses to anandamide were measured on top of Tocrisolve™. Anandamide failed to evoke substantial effect on control fetal cerebral arteries (n=4), with the change in an artery diameter in presence of 10 µM anandamide reaching −0.83 ± 1.19% (Figure 2C, E). In arteries from alcohol-exposed fetuses (n=4), we observed large vasodilatory responses to anandamide (approximately 8 and 15% dilation) on two occasions. These responses shifted the average change in artery diameter to 5.17 ± 4.16%. The difference between control and alcohol-exposed groups did not reach statistical significance (P=0.49 by Mann-Whitney test; Figure 2E).

Consistent with data from fetal arteries, maternal arteries from the control group (n=2) failed to dilate in presence of 10 µM anandamide: change in an artery diameter was 1.72 ± 0.35% (Figure 2C–E). In arteries from alcohol-exposed mothers (n=4), on two occasions we observed the large vasodilatory responses to anandamide (17 and 21% dilation) similar to the vasodilatory responses observed in fetuses. A large vasodilation was observed in arteries from a mother-fetus pair. However, in another pair, vasodilation was observed in the maternal, but not the corresponding fetal vessel. The vasodilatory responses in maternal arteries shifted the average change in an artery diameter to 9.92 ± 5.33%. The difference between control and alcohol-exposed maternal arteries did not reach statistical significance (P=0.53 by Mann-Whitney test; P=0.3629 by unpaired t-test; Figure 2E). The two-way ANOVA failed to detect any interaction between age or alcohol exposure variances (P≥0.12).

**Figure 3:**
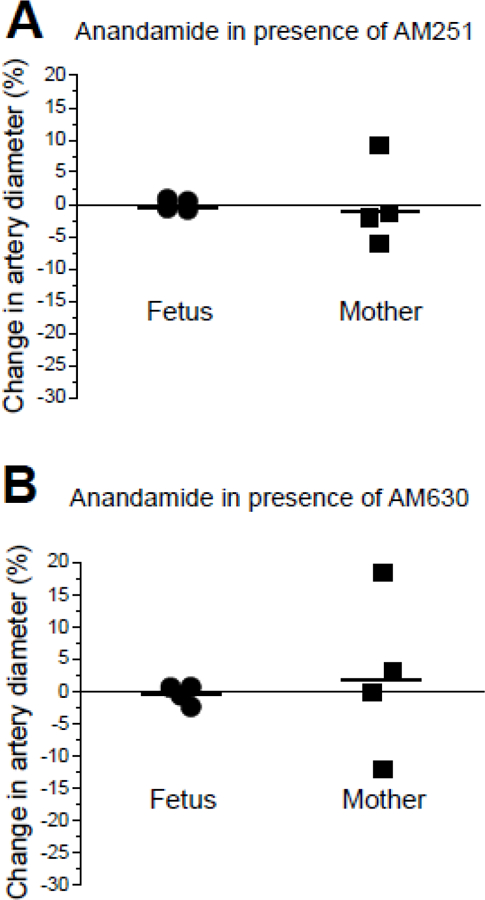
Anandamide-induced changes in artery diameter of alcohol-exposed fetal and maternal cerebral arteries in presence of CB receptors’ blockers. A. Scattered data of changes in artery diameter by 10 µM anandamide in fetal and maternal arteries from alcohol-exposed donors in presence of CB1 receptor blocker 1 µM AM251. B. Scattered data of changes in artery diameter by 10 µM anandamide in fetal and maternal arteries from alcohol-exposed donors in presence of CB2 receptor blocker 1 µM AM630.

We decided to further investigate the apparent lack of difference between fetal and maternal artery response to anandamide, as age-dependent changes were reported for the eCB system (reviewed by [20–21]). Considering the existence of apparent vasodilatory responses in alcohol-exposed fetal and maternal arteries in presence of anandamide, we focused on alcohol-exposed groups and set to determine whether the anandamide-driven vasodilation was mediated by any of the canonical CB receptors (CB1 and CB2). Testing of 10 µM anandamide in presence of CB1 receptor blocker 1 µM AM251 [22] did not yield any measurable vasodilatory responses in the arteries from alcohol-exposed fetuses (n=4); the average change in an artery diameter was −0.03±0.40% (Figure 3A). Remarkably, in maternal arteries from the alcohol-exposed group (n=4), 9% vasodilation was still observed in presence of AM251 (Figure 3A); the average change in an artery diameter reached −0.08 ± 3.26%.

Testing of 10 µM anandamide in presence of CB2 receptor blocker 1 µM AM630 [23–24] did not yield any measurable vasodilatory responses in the arteries from alcohol-exposed fetuses (n=4); the average change in an artery diameter was −0.46 ± 0.72% (Figure 3B). However, in maternal arteries from the alcohol-exposed group (n=4), 18% vasodilation was still observed in presence of AM630 (Figure 3B); the average change in an artery diameter reached 2.29 ± 6.28%. The qualitatively different effect of CB receptor block on fetal and maternal arteries suggests the existence of age-dependent differences in eCB signaling between fetal and maternal vessels.

### 3.3. Alcohol modulation of a cerebral artery diameter is modified during development

**Figure 4:**
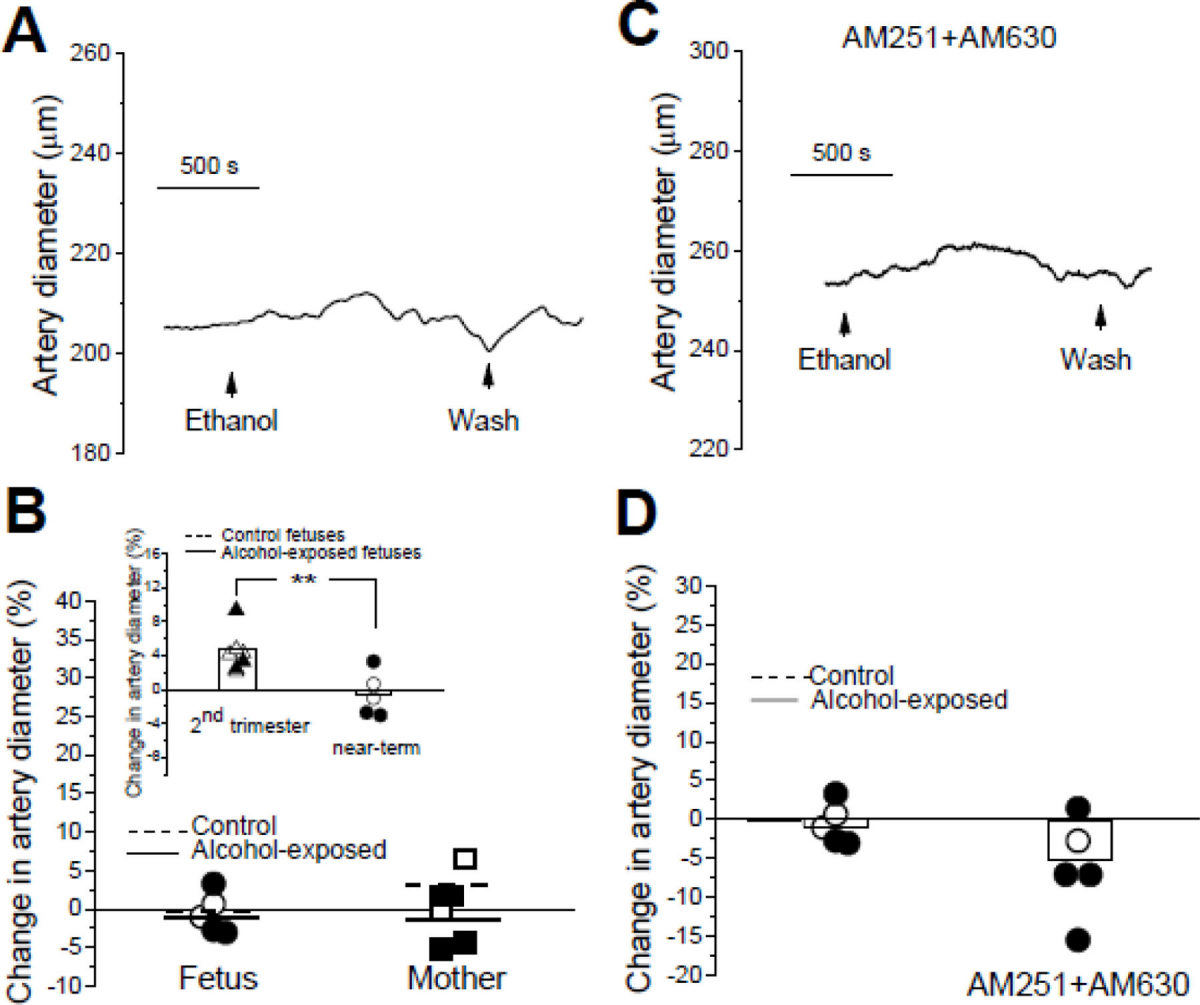
Ethanol effect on cerebral artery diameter is modified during gestation. A. Original record of diameter showing lack of ethanol-induced dilation in cerebral artery from near-term fetus. B. Scattered data of changes in artery diameter by 63 mg/dL ethanol in fetal and maternal arteries. Statistical analysis using two-way ANOVA failed to detect significant differences or interactions between the groups at the 0.05 level (P values≥0.28). Insert depicts statistically significant difference in ethanol-induced change of artery diameter between arteries harvested at the end of the period equivalent to the second trimester of human pregnancy and at near-term. In the insert, “2^nd^ trimester” data were obtained in the course of our previous study [8], while “near-term” represents data from current work. In the insert, hollow columns depict averaged data from control and alcohol-exposed fetuses at the end of 2^nd^ trimester equivalent, and averaged data from control and alcohol-exposed fetuses near-term. *Statistically significant difference, P=0.0072 (unpaired t-test). C. Original trace showing effect of 63 mg/dL ethanol on near-term fetal cerebral artery dimeter in presence of AM251 and AM630 mixture. D. Scattered data of changes in diameter of near-term fetal cerebral arteries by 63 mg/dL ethanol in absence versus presence of AM251 and AM630 mixture. Hollow columns represent averaged data from maternal and fetal arteries near-term in absence versus presence of CB receptor blockers.

We have recently shown that 63 mg/dL ethanol (that is an alcohol concentration found in amniotic fluid of pregnant baboons under alcohol intoxication) dilates fetal cerebral arteries that are harvested from fetuses at the end of the second trimester equivalent [8]. To determine whether ethanol dilates cerebral arteries at later time-points, we probed *in vitro* pressurized cerebral arteries of fetuses and their mothers with 63 mg/dL ethanol. Ethanol largely failed to evoke an effect on the control fetal cerebral arteries (n=2), with the change in an artery diameter in presence of 63 mg/dL ethanol reaching −0.20±0.85% (Figure 4A–B). In the arteries from alcohol-exposed fetuses (n=3), the average change in an artery diameter was −0.83 ± 2.06%. The difference between control and alcohol-exposed groups did not reach statistical significance (P=0.80 by Mann-Whitney test; P=0.8328 by unpaired t-test; Figure 4B). In maternal arteries from the control group (n=2), the ethanol-induced change in an artery diameter in presence of 63 mg/dL ethanol reached on average 3.11 ± 3.37% (Figure 4B). In the arteries from alcohol-expose mothers (n=4), the average change in an artery diameter by ethanol was −1.58 ± 1.89%. The difference between control and alcohol-exposed groups did not reach statistical significance (P=0.53 by Mann-Whitney test; P=0.2519 by unpaired t-test; Figure 4B).

The lack of consistent ethanol-induced dilation in our experiments prompted us to compare the ethanol responses of fetal arteries harvested from these near-term donors with the ethanol responses that we obtained earlier in cerebral arteries from fetuses at the end of the second trimester equivalent [8]. Considering that at either gestational stage there was no statistically significant difference in ethanol responses between control and alcohol-exposed groups, the two groups were treated as a single dataset for the purpose of comparing ethanol responses of *in vitro* pressurized arteries. In both, the mid-pregnancy (n=7) and near-term groups (n=5), datasets followed a Gaussian distribution. The average ethanol-induced dilation in fetal arteries harvested at the end of the mid-pregnancy reached 4.33 ± 0.91% whereas in a near-term group the ethanol effect was lost. Changes in an artery diameter in presence of 63 mg/dL in this group averaged −0.58 ± 1.17% (Figure 4B, insert). The difference between two groups was statistically significant (P=0.0072 by the unpaired t-test). Our data document that the ability of ethanol to dilate fetal cerebral arteries is limited to a particular gestational time and is vanishing near-term.

As we have previously established, alcohol-induced dilation of *in vitro* pressurized cerebral arteries probed at the end of mid-pregnancy was totally blunted in presence of CB1 and CB2 receptors blocker mixture [8]. In the present work, we set to determine whether CB receptors block would modify the ethanol effect on fetal cerebral arteries harvested near-term. Considering that maternal and fetal cerebral arteries near-term did not differ in their sensitivity to ethanol, these arteries were treated as a single dataset for the purpose of comparing artery diameter when ethanol was probed in absence versus presence of CB receptor blockers. Consistent with the lack of ethanol-induced dilation in arteries from near-term fetuses (Figure 4A, B insert), a pharmacological block of CB1 and CB2 receptors at this gestational age did not significantly modify ethanol responses of the artery (Figure 4C–D). To compare ethanol effect on a cerebral artery diameter in the absence versus presence of CB1 and CB2 receptors blockers, we treated arteries from control and alcohol-exposed fetuses at each experimental condition as a single dataset. The average change in an artery diameter by 63 mg/dL ethanol in presence of AM251 and AM630 blockers reached −6.22 ± 2.81% (n=5) and did not differ significantly from ethanol effect without blockers (−0.58 ± 1.17%, n=5; P=0.10 by the unpaired t-test). Thus, cross-talk between eCB system and ethanol in control of a cerebral artery diameter is limited to a particular gestational time and is minimal, if any, near-term.

## 4. Discussion

In the present work, we obtained several novel findings using a baboon model of pregnancy. First, we established that *in vitro* pressurized cerebral arteries harvested from near-term fetuses have an ability to develop tone and to respond to a non-specific contractile agent (high KCl) in a matter that is similar to responses of maternal arteries. With regard to modifications of a cerebral artery diameter by toxicologically relevant levels of alcohol, we documented that the up-regulation of fetal cerebral artery CB2 receptor function is limited to mid-pregnancy. This up-regulation was not observed at near-term. Lastly, we documented that ethanol modification of a fetal cerebral artery diameter depends on the gestational age of the fetuses. In particular, CB receptor-mediated dilation of fetal cerebral arteries by ethanol that was shown to take place during mid-gestation [8], was not present near-term, and the contribution of CB receptors to ethanol effect on a cerebral artery diameter was lost.

The fact that near-term fetal cerebral arteries of baboon exhibited consistent autoregulation (myogenic tone) and constriction in response to KCl similar to those of adult maternal arteries (Figure 1) was quite surprising, as perinatal cerebral circulation in other species, including humans, often exhibits higher inter-individual variability and vulnerability to stressors when compared to that of adult individuals [25]. Higher variability in fetal organ responses to pharmacological modulators is attributed to the complex course of developmental curves of proteins and signaling molecules that control myogenic tone in cerebral arteries [26,27,28,29]. Interestingly, we detected a qualitative difference in KCl-induced constriction of fetal arteries when compared to maternal ones. Indeed, in maternal arteries, the KCl effect was often biphasic, with subtle dilation preceding a development of constriction (Figure 1C). The similar biphasic effect of 60 mM KCl was observed in other species [18,19] and could be attributed to the vasodilatory effect of low KCl concentrations that was reported in coronary and cerebral arteries [30]. In a pressurized artery technique, experimental solutions are introduced into an artery perfusion chamber gradually; it takes several minutes to substitute physiologic saline with high KCl solution. Thus, the initial phase of KCl effect, i.e., vasodilation, could be explained by low KCl targeting inward rectifier K^+^ channels [30]. The lack of biphasic responses to KCl presence in near-term fetal arteries suggests the lack of inward rectifier K^+^ channel involvement into an artery diameter regulation at this gestational stage.

With regard to the eCB system, it has been widely recognized as a critical player in fetal development [31,32]. Recently, we have detected up-regulation of vasodilatory responses to anandamide when probed *in vitro* at mid-pregnancy following three repeated episodes of maternal alcohol intoxication [8]. The current study aimed to determine whether up-regulation of vasodilatory responses of fetal cerebral arteries to anandamide was long lasting and could be observed near-term following repeated episodes of maternal alcohol intoxication during the mid-pregnancy (Material and Methods). We did not detect statistically significant differences in anandamide-driven changes of a cerebral artery diameter between near-term fetal and maternal arteries (Figure 2). This result suggests that the alcohol-triggered modification in cerebral artery response to anandamide that we observed previously [8] is transient. Notably, the transient nature of alcohol-driven re-shaping of eCB system components has been recently reported in male rat entorhinal cortex and hippocampus [33]. In contrast, another recent study has documented life-long changes in arterial function following prenatal (fetal) alcohol exposure [7]. In particular, fetal alcohol exposure alters age-dependent changes in blood velocity within carotid artery and impairs post-stroke behavioral recovery in adult mice [7]. Thus, besides transient changes in fetal artery eCB system, alcohol is expected to target other signaling pathways that result in permanent changes in arterial function. The mechanisms that underlie the transient nature of alcohol-driven re-shaping of eCB system components remain unknown and may range from de-sensitization of relevant alcohol targets to the development of compensatory mechanisms that negate alcohol presence.

At the level of fetal cerebral circulation, alcohol at physiologically relevant levels (<300–400 mg/dL; [13, 34]) was consistently reported to increase fetal cerebral perfusion or, accordingly, drop fetal cerebral artery Doppler indices of cerebral blood flow velocity [5,6,10,11]. Both outcomes are consistent with dilation of fetal cerebral arteries. Indeed, we have recently shown that ethanol at concentration that is reached in amniotic fluid during alcohol intoxication in pregnant baboons, dilated *in vitro* pressurized cerebral arteries harvested from fetuses at the end of the second trimester equivalent [8]. In the present work, however, fetal cerebral artery dilation by ethanol vanished later in gestation, as ethanol-driven changes in a fetal cerebral artery diameter near-term (Figure 4A-B) were significantly smaller than those changes in fetal cerebral arteries harvested at the end of the mid-pregnancy (Figure 4B insert; [8]). In addition, there was no apparent dilation by ethanol in adult maternal cerebral arteries (Figure 4B). The lack of ethanol-induced dilation during the perinatal period and adulthood is consistent with other reports that describe ethanol-induced constriction of adult cerebral arteries harvested from rodent species and dogs [18,19, 35].

Reasons for the age-dependent effect of ethanol remain speculative. However, the most straightforward explanation would be the loss of relevant ethanol targets that were present during mid-pregnancy and/or emerging of new targets during pre-term stage of development. Indeed, ethanol-induced dilation of fetal cerebral arteries during mid-pregnancy was totally blunted by a mixture of CB1 and CB2 receptor blockers. Moreover, ethanol-induced dilation was turned into a measurable constriction when CB receptors were blocked [8]. To some extent, similar phenomenon (i.e. ethanol-induced constriction in presence of CB receptor blockers) was observed in several artery segments in the current work (Figure 4D). This phenomenon may be explained by the involvement of calcium-/voltage-gated potassium channel of large conductance (BK) in artery diameter regulation by ethanol. Indeed, in adult cerebral arteries, ethanol-induced constriction was shown to arise from ethanol-induced inhibition of BK channels [18]. More recently, ryanodine receptors (RyR) were also identified as highly-sensitive proteins that control cerebral myocyte contractility [37]. Notably, RyR function was shown to be similar in ovine fetal and adult cerebral arteries [27], while BK channels evolve during development [29,38]. Thus, BK channels may emerge as a relevant ethanol target at late gestational stage. The idea that eCB system within a fetal cerebral artery could lose functional significance after birth seems plausible considering that age-dependent modifications in the eCB system have been documented in several reports [39,40].

We would like to note that our study has several limitations. These limitations stem from the experimental model – non-human primates. Undoubtedly, non-human primates (including baboons) offer an invaluable advantage over other widely used laboratory animals for studies on PAE and fetal cerebral artery (reviewed by [9]). Indeed, baboon reproductive physiology and obstetrics closely resemble those of human [41]. Moreover, non-human primate overall physiological and behavioral similarities with humans make them an attractive model for alcohol researchers [42]. However, the availability of primates for experimental work is limited. Thus, sample sizes in our work are very small, and this limitation raises the issue of false-positive as well as false-negative discoveries [17]. However, several pieces of our current data point at the lack of long-lasting effect of PAE during mid-pregnancy on fetal cerebral artery eCB system. It is unlikely that such consistency could be reached with false discoveries. Another limitation is the baboons’ heterogeneous medical histories and environmental exposures. Although this heterogeneity closely resembles the diversity of human population, it also introduces variability in experimental outcomes. Thus, our study is likely underpowered to detect subtle differences between the groups.

## 5. Conclusions

Our data document that modifications in fetal cerebral artery eCB system triggered by fetal alcohol exposure during mid-pregnancy are transient as they are not detected near-term. Moreover, ethanol-induced dilation of fetal cerebral arteries is characteristic of a specific gestational age and disappears later during gestation. Considering these findings, we conclude that eCB system contribution into ethanol targeting of the fetal cerebral artery diameter is limited to mid-pregnancy. Moreover, the effect of PAE on CB2 receptor sensitivity to anandamide is not long-lasting. Future studies will determine the role of the transient changes in fetal cerebral artery eCB system that are induced by fetal alcohol exposure in the development of FASDs abnormalities.

## Significance.

Our findings point at a gestational stage-dependent effect of ethanol on fetal cerebral artery and thus, contribute to our understanding of FASD pathophysiology.

## Abbreviations:

AEA: Anandamide
BAL: Blood Alcohol Level
BK: calcium-/voltage-gated potassium channel of large conductance
CB1: Cannabinoid (receptor) type 1
CB2: Cannabinoid (receptor) type 2
eCB: Endocannabinoid (system)
FASDs: Fetal Alcohol Spectrum Disorders
MCA: Middle Cerebral Artery
PAE: Prenatal Alcohol Exposure
PSS: Physiologic Sodium Saline
RyR: Ryanodine Receptor
